# Revolutionizing pig farming: Japan’s technological innovations and environmental strategies for sustainability

**DOI:** 10.5455/javar.2025.l912

**Published:** 2025-06-16

**Authors:** Md Kamrul Hasan, Hong-Seok Mun, Eddiemar B. Lagua, Hae-Rang Park, Young-Hwa Kim, Md Sharifuzzaman, Jin-Gu Kang, Chul-Ju Yang

**Affiliations:** 1 Animal Nutrition and Feed Science Laboratory, Department of Animal Science and Technology, Sunchon National University, Suncheon, Republic of Korea; 2 Department of Poultry Science, Sylhet Agricultural University, Sylhet, Bangladesh; 3 Department of Multimedia Engineering, Sunchon National University, Suncheon, Republic of Korea; 4 Interdisciplinary Program in IT-Bio Convergence System (BK21 Plus), Sunchon National University, Suncheon, Republic of Korea; 5 Department of Animal Science and Veterinary Medicine, Gopalganj Science and Technology University, Gopalganj, Bangladesh; † These two authors contributed equally to this work.

**Keywords:** Japan, pig farming, precision technology, animal welfare, sustainability.

## Abstract

**Objective:**

This review examines Japan’s pig farming landscape, highlighting key barriers while exploring projects that foster large-scale sustainable development efforts by emphasizing precision technologies integration and policy implications.

**Materials and Methods:**

A literature review was conducted using keyword searches across Google Scholar, covering studies published between 2018 and 2024. The review encompassed studies on Japan’s pig farming, addressing prospects, production metrics, challenges, consumption patterns, market trends, precision technologies, and insights from peer-reviewed journals, credible websites, government reports, and conference proceedings.

**Results:**

Japan, one of Asia’s largest pork consumers, relies on imports, with domestic production covering only 47.08% of consumption, highlighting a need for greater efficiency. Although small-scale farms continue to dominate the pig industry, the sector is navigating a pivotal shift toward modernization and the expansion of large-scale operations. Farmers face mounting pressures from feed costs, labor shortages, diseases, and strict environmental regulations. Precision pig farming technologies address these by optimizing resource use, enabling early disease detection to reduce costs, improving herd health to promote better welfare, and managing manure to reduce emissions.

**Conclusion:**

Integrating large-scale operations with precision pig farming technologies can redefine Japanese pig farming, promoting animal welfare and environmental sustainability. The government must secure financial backing (partial or full subsidies) to support large-scale operations, tax reductions on imported tools, and grants to foster domestic tools and renewable energy innovations to achieve this. Future life-cycle assessment research will be essential for evaluating the long-term environmental impacts, ensuring viability, and promoting sustainability in Japan’s pork production sector.

## Introduction

The global pork market, which was valued at 254.53 billion USD in 2022, is expected to grow by approximately 8.64% annually, reaching 418.37 billion USD by 2028 [1]. Asia’s main markets for pork consumption are the Republic of Korea, Taiwan, and Japan [2]. Per capita pork consumption in Japan is increasing every year [3]; however, its domestic production only meets about 47.08% of total consumption [4]. By spending 3.9 billion USD on pork imports in 2023, Japan solidified its status as a leading global pork importer [5]. The number of pig farms has been decreasing; however, the number of pigs per farm has increased [4].

Therefore, in Japan’s large-scale pig farms, maintaining productivity and improving management for disease prevention have taken precedence [6]. Environmental issues, for example, nutrient leaching [7], pollutant runoff [8], and heavy metal accumulation in soils [9], are exacerbated by large-scale pig farming, which produces excessive amounts of manure [10]. This emphasizes how urgently creative solutions are needed to reduce these risks and enhance farm management. Precision livestock farming (PLF) can be a crucial part of the optimal farm management strategy, which can improve the sustainability, health, and efficiency of pig farming operations. PLF integrates engineering principles through the use of PLF tools (sensors and devices) to enable real-time, automated monitoring of livestock farms [11]. A subset of PLF known as precision pig farming uses cameras, accelerometers, sensors, the Internet of Things (IoT), and other digital technologies in farms to track health and welfare in real-time, as well as the environment, precision feeding, and waste disposal [12–16]. This early detection of health, welfare, and environmental problems may assist farmers in deciding on efficient farm management.

Japan’s pig farming sector struggles with farm management due to labor shortages [17], aging farmers [18], high production costs [4], and rising consumer demand for quality pork [19]. To address labor shortages in the long term, farmers are expressing interest in using PLF tools [20]. Japan is one of the technologically advanced countries, especially in the field of artificial intelligence (AI) and robotics [21]. Recently, various types of PLF technology have advanced; for example, e-kakashi and e-kakashi Tetori can assist farmers in monitoring and optimizing the environmental conditions of barns [22]. To meet the growing domestic pork demand, farm sizes are increasingly shifting toward large-scale operations [23], a trend that makes it nearly impossible for farmers to monitor individual animals effectively.

Incorporating PLF in large-scale farming may provide an opportunity to achieve sustainable pig production by enhancing efficiency and reducing resource consumption. While Japan has made significant strides in PLF adoption within its dairy (90%) and beef cattle (69%) farms, pig farming lags, with only 50% of farms integrating these technologies [24]. Despite Japan’s advanced PLF capabilities [22], research on their role in sustainable large-scale pig farming is limited, and adoption lags behind other developed nations [25]. Therefore, there is an urgent need to identify the challenges hindering pig farming, as well as to explore strategies that could expedite the uptake of technologies for precision pig farming, which would ultimately enhance farm management and support farmers in achieving sustainable production. Addressing these barriers is critical not only to improving farm productivity but also to ensuring the long-term viability of the industry by optimizing resource use and minimizing environmental impact.

By meticulously scrutinizing the existing literature, this study examines the challenges and factors influencing the advancement of pig farming in Japan. The objective is to explore how precision pig farming technologies can address issues related to pig farming and how farmers can incorporate precision technologies in large-scale farming. By assessing the current state of technologies for precision pig farming and their impact on farm management, this study seeks to identify strategies that optimize pig production, improve animal health and welfare, and enhance sustainability.

## Materials and Methods

### Searching and selecting articles

A literature review of relevant articles was conducted. First, the exclusion and inclusion criteria were set, keywords were defined, and academic and non-academic databases were selected for the publication search. Subsequently, the articles were then filtered to determine which ones were most relevant to the current study. The aim was to draw conclusions and suggest measures to foster sustainable precision pig farming. A combination of different keywords—”large-scale,” “pig farming,” “Japan,” and “precision”—was used for searching articles from Google Scholar over the last 7 years (2018–2024). The Boolean operator “AND” was used with these keywords to improve literature searches, and the results yielded an initial pool of 248 papers. To ensure the selected literature’s relevance, a meticulous screening of titles, abstracts, and keywords led to the exclusion of 203 papers misaligned with the study’s core focus. Subsequently, a thorough content analysis and full-text assessment scrutinized the remaining articles for insights into Japan’s pig-farming challenges and emerging trends.

**Figure 1.**
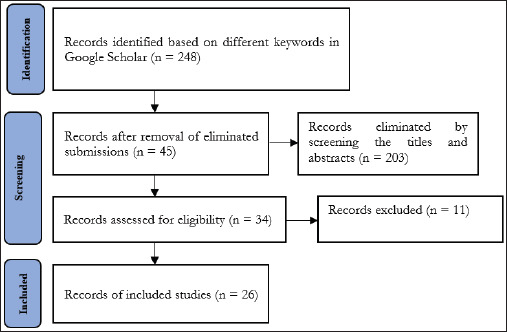
PRISMA flowchart for choosing papers.

As a result, the number of relevant studies was further narrowed down to 34. To strengthen the review’s rigor and minimize the risk of missing critical insights, each of the 34 articles was individually assessed for thematic scope, research depth, and future implications. Following a rigorous evaluation, 26 studies were identified as most relevant and retained for in-depth analysis. The selection process was systematically organized and visually depicted through the PRISMA flowchart (Fig. 1). The search was expanded to include non-academic databases, reports from international projects, official government papers, policy documents, and reliable news sources in Japanese and English to cover current cases of precision pig farming in Japan. The quality of non-peer-reviewed sources was assessed using the credibility, accuracy, reasonableness, and support (CARS) checklist.

## Inclusion criteria

- Studies discussed the challenges in Japan’s pig farming, government policies, and technological advances in precision farming.
- Data on pig production, marketing, and consumption in quantitative terms.
- Articles included worldwide precision farming techniques in livestock production, with a particular focus on pig farming.
- Sources comprised peer-reviewed academic journals, websites of Japanese ministries, government, and international reports, and reliable non-academic materials in both Japanese and English. Any Japanese content was translated into English using Google Translate.

## Exclusion criteria

- To concentrate on the latest technological and policy developments, articles published before 2018 were excluded.
- The review excluded articles focused on non-pig farming systems, such as poultry, cattle, or crop farming.
- Publications in languages other than English and Japanese, and those lacking credibility or depth, were excluded.

## Data acquisition and statistical analysis

Information from selected literature, government and international reports, and websites of Japanese ministries, encompassing charts and text, was curated and structured in Microsoft Excel to enable tabular and visual representations, aiding in discerning patterns, challenges, opportunities, and evolving trends. SPSS Statistics 17.0 was utilized for the correlation analysis of pig production cost with parameters related to the economics and market dynamics of the pig industry, with significant differences at a 5% level.

## Pig production and market dynamics

**Figure 2.**
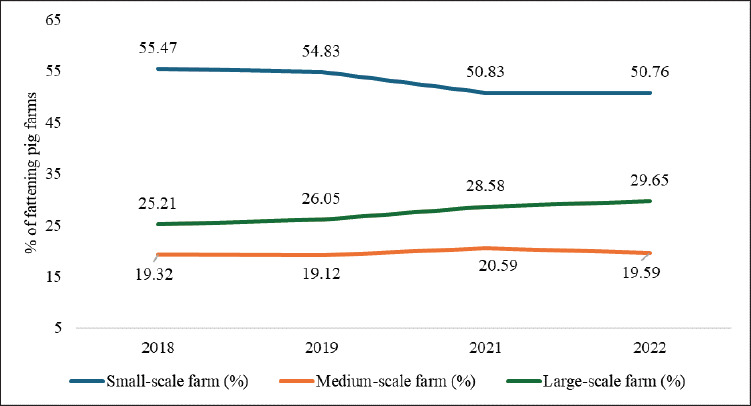
Trends in Japanese fattening pig farms by scale (2018–2022). Small-scale farms (<1,000 pigs) declined from 55.47% to 50.76%, while large-scale farms (≥ 2,000 pigs) grew from 25.21% to 29.65%, signaling industry consolidation. Medium-scale farms (1,000–1,999 pigs), remained stable, reflecting structural shifts favoring larger operations. Generated from [23].

**Figure 3.**
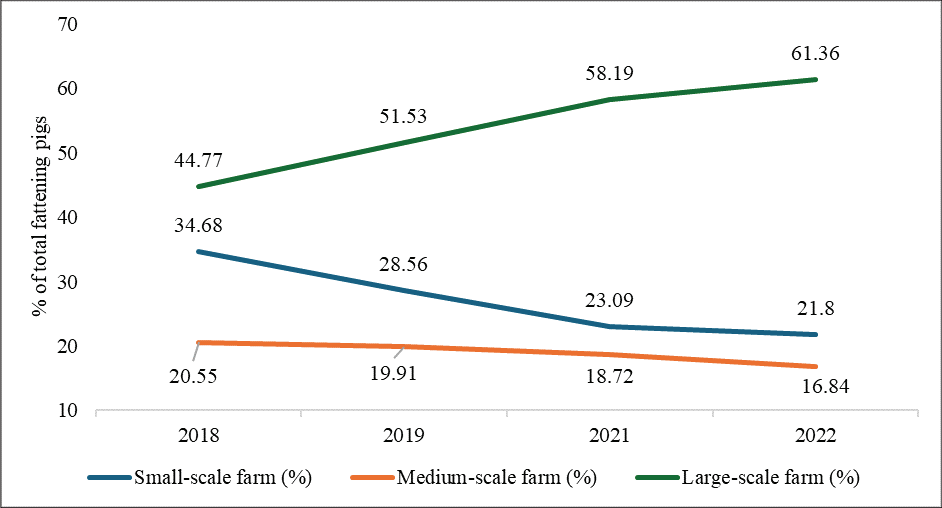
Contribution of Japanese fattening pig farms by scale to total pig production (2018–2022). Large-scale farms (≥ 2,000 pigs) expanded their dominance, increasing from 44.77% to 61.36%, while small-scale farms (< 1,000 pigs) declined from 34.68% to 21.8%, underscoring industry consolidation. Medium-scale farms (1,000–1,999 pigs) showed a slight decrease, reflecting a structural shift favoring high-capacity operations. Generated from [23].

Japan’s pig farming has traditionally been dominated by small-scale farms (<1,000 pigs), approximately 55.47% in 2018, but declined to 50.76% by 2022 (Fig. 2) [23], driven by high production costs [26], labor shortages [17], and stricter environmental [27] and animal welfare regulations [28]. Many small-scale farmers have transitioned to medium (1,000–1,999 pigs) and large-scale operations (≥2,000 pigs), which offer higher profitability [29]. Medium and large-scale operations reduce feed, labor, and production costs relative to small-scale operations [30]. Large-scale farms exhibit better productivity than smallscale, for example, annual litters per sow (2.36 vs. 2.24), piglets per litter (12.76 vs. 11.96), and piglets weaned annually (26.34 vs. 23.85) [31]. The contribution of largescale fattening farms to the total number of fattening pigs increased to 61.36% in 2022, reflecting a 37.04% rise since 2018 (Fig. 3) [23].

**Figure 4.**
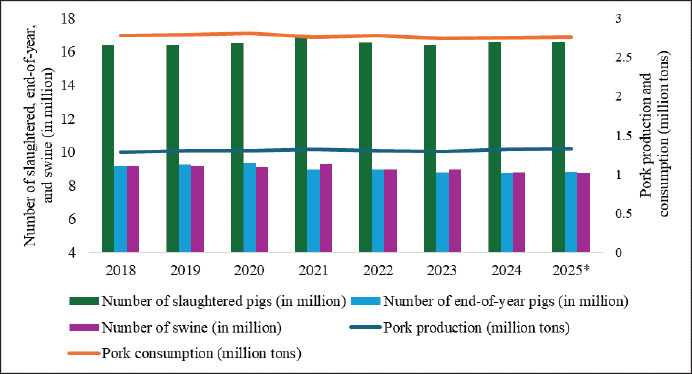
Trends in Japan’s pig industry (2018–2024) with a 2025 forecast. Slaughter rates and end-of-year pig stocks remained stable, while pork production showed a slight upward trend. Despite this, pork consumption consistently exceeded domestic production, indicating reliance on imports [4,32,33]. * Predicted value for 2025 [4].

**Figure 5.**
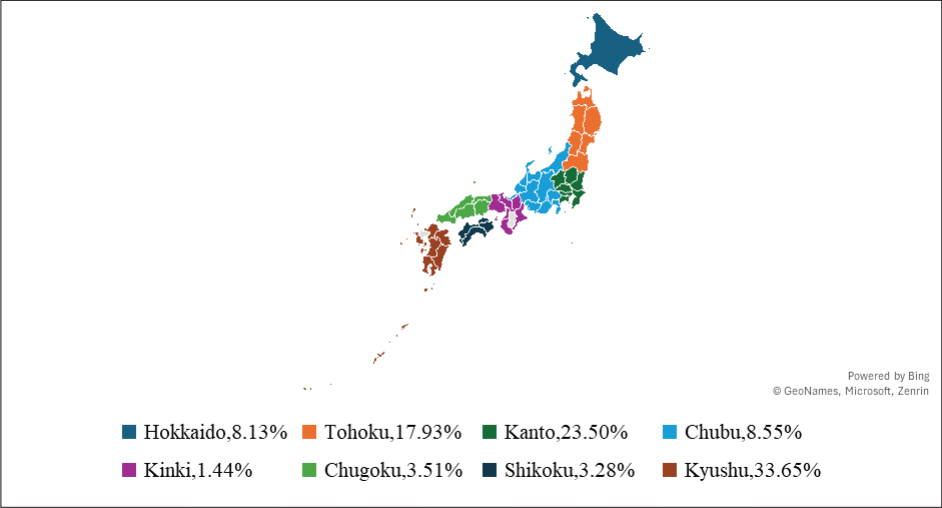
Regional distribution of Japanese pig population in 2022. The Kyushu region, accounting for 33.65% of the pig population, stands as the dominant hub of pig production, while the Kinki region lags behind with only 1.44%, revealing significant regional disparities in pig farming concentration across the country. Generated from [23].

The trend of decreasing small-scale farms (Fig. 2) [23] has contributed to a decline in the total pig population, from 9.19 million in 2018 to an estimated 8.79 million in 2024, while slaughter numbers remained stable at 16.43–16.60 million (2018–2024) (Fig. 4) [4,32,33], due to the efficiency of large-scale farms. Regional disparities are significant in 2022, with Kyushu hosting one-third of the pig population, driven by its subtropical climate (15°C–19°C) [34] and a shift from rice cultivation to livestock farming [35], whereas urbanization and land scarcity may limit the Kinki region to only 1.44% (Fig. 5) [23].

**Figure 6.**
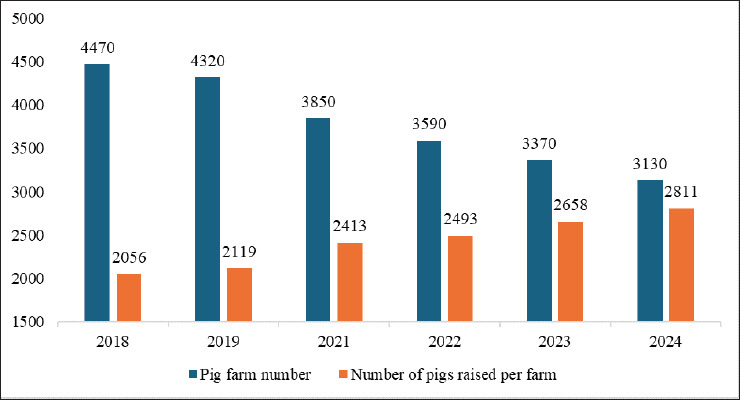
Pig farm numbers and pigs raised per farm in Japan (2018–2019 and 2021–2024) [4]. The number of pig farms has declined from 4,470 in 2018 to 3,130 in 2024, signaling consolidation within the industry. Meanwhile, the average number of pigs per farm has increased from 2,056 to 2,811, reflecting growing efficiency and intensification in farming practices.

An approximately 36.7% rise in the number of pigs per farm from 2018 to 2024 (Fig. 6) [4] highlights the trend toward larger-scale farming. By 2022, these farms accounted for 29.65% of all operations, a 4.44% rise since 2018 (Fig. 2) [23]. Of these farms, 65% are operated by companies [36], offering higher economic benefits [29] despite persistent waste management concerns [37]. To address environmental challenges, PLF technologies have been introduced [38], supported by government loans and subsidies only for hilly-area farmers [39]. Despite the increased efficiency of large-scale farms, domestic pork production has stagnated between 1.28 and 1.32 million tons (2018–2024), falling short of consumption, which was 2.13 times higher in 2023 (Fig. 4) [4,32,33]. To bridge this supply gap, imports from the USA, Canada, Spain, and Mexico play a crucial role [40], with Japan allocating $3.93 billion to pork imports in 2023, 80.87% of which went to the USA ($982.4 million), Canada ($954 million), Spain ($697 million), and Mexico ($544.7 million) [5]. Alongside imports, leading domestic producers including NH Foods Ltd., Global Pig Farms Inc., Yamagata Ham Co., Ltd., Daisui Co., Ltd., and Mitsui & Co., Ltd., supply local markets, catering to consumer demand with indigenous breeds, for example, Kagoshima Berkshire, Waton Mochibuta, and the Japanese Native Pig (Nihon Genshoku Buta) [41,42].

## Challenges in pig farming

### Land

With three-fourths of the land covered by mountains [43], the country faces a severe shortage of arable land, providing only 0.03 hectares of arable land per capita in 2021 [44]. Urbanization has intensified land strain [45], reducing arable land by 1.35% between 2018 and 2021 [44]. These challenges are particularly acute for livestock farming, which demands more space than crop cultivation [46,47], making it increasingly difficult to sustain conventional farm operations. Vertical farming can be an innovative solution to these constraints, maximizing space efficiency and integrating seamlessly with urban areas [48]. Large-scale multi-floor pig farming systems reduce greenhouse gas (GHG) emissions by 30%, minimize land use for breeding by 91%, and lower labor requirements by 72% compared to conventional systems, enhancing efficiency and sustainability [49].

### Labor

**Figure 7.**
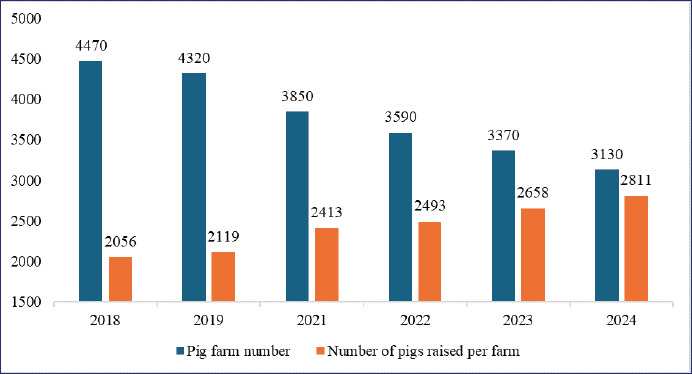
Feed, labor, medicinal, other, and production costs, as well as sale prices of pigs in Japan (Fiscal Year 2018–2022) [30]. There was an increasing trend in production costs, primarily driven by rising feed expenses, which surged from 20,500 JPY in FY 2018 to 29,300 JPY in FY 2022. Labor, medicinal, and other expenses, also grew moderately. While sale prices fluctuated over the years, the gap between production cost and sale price became critical in 2022, when the production cost per pig (43,700 JPY ) exceeded the sale price (41,202 JPY), resulting in a loss of 2,498 JPY per pig, highlighting the growing financial strain on Japan’s pig farming sector.

The decline in pig farming stems from labor shortages, rising labor costs, and an aging farming population. Between 2020 and 2024, the population decreased by 2.09% [50], exacerbating rural labor [51]. Labor costs per pig rose by 15.91%, from 4,600 JPY in fiscal year (FY, from April to March) 2018 to 5,100 JPY in FY 2022, increasing production costs (Fig. 7) [30]. The aging population, with 29% of individuals aged ≥ 65 years in 2022, nearly three times the global average, further weakens the agricultural workforce [52]. This demographic shift is projected to worsen, with the proportion of people aged ≥ 65 years expected to rise approximately 38.4% by 2065 [53]. To mitigate labor shortages, Japan developed an autonomous cleaning robot that enhances efficiency, reducing pig farm sanitation time by 66% to 68% relative to manual labor [54]. However, more innovative solutions are needed for sustainable pig production.

### Feed

Feed costs represent a significant portion of pig production expenses, accounting for 60% to 70% of the total cost [27]. From FY 2018 to FY 2022, feed costs increased substantially, rising from 61.75% to 67.05% of the total production cost (Fig. 7) [30]. In FY 2020, when the feed cost per pig was JPY 20,300, the overall pig production cost stood at JPY 33,900 per pig, allowing farms to achieve a relatively higher profit of JPY 5,300 per pig (Fig. 7). However, as the feed cost per pig began to increase in FY 2021, the production cost surged alongside it, eroding farm profitability, with no profits during that period. By FY 2022, when the feed cost reached a peak of JPY 29,300 per pig, farmers faced a significant loss of JPY 2,498 per pig (Fig. 7). This dramatic rise in feed costs ultimately made pig farming increasingly challenging for farmers, as it inflated production costs and reduced their ability to generate profits, highlighting the vulnerability of the industry to surges in feed prices. This surge can be attributed to a 50% rise in compound feed prices, driven by higher costs of raw materials, for example, corn [55]. Rising feed prices drove a 42.93% increase in feed costs per pig, amounting to an additional 8,800 JPY between FY 2018 and FY 2022 (Fig. 7) [30], intensifying financial pressure on farmers. The reliance on imported corn makes pig feed approximately twice as expensive as in the USA, further exacerbating the situation [55]. The government is working to reduce dependency on imported feed by increasing subsidies for rice cultivation and exploring replacing corn with rice in livestock feed [55]. Initiatives, for example, feed funds, are being developed to support farmers in cultivating feed ingredients. Moreover, meat and food companies are mandated to use domestically produced feed ingredients. The government aims to increase the stagnant 25% self-sufficiency rate for animal feed to 34% by 2030 through support for local farmers and sustainable feed development [55]. Reducing feed costs through domestic production remains a significant challenge, requiring innovation to make local feed ingredients more cost-effective.

### Disease

Classical swine fever (CSF) and porcine reproductive and respiratory syndrome (PRRS) continue to challenge pig farming, with the CSF virus reemerging in 2018 [56] after being declared CSF-free in 2007 [57], spreading despite immediate control measures, for example, culling, movement restrictions, and vaccination efforts. By 2023, the outbreaks had led to the culling of 368,000 pigs across 20 prefectures [58], and in May 2024, significant outbreaks in Hirono-Town, Iwate Prefecture, affected over 17,500 pigs [59]. The resurgence is largely driven by wild boar populations, which act as virus reservoirs and complicate containment efforts [60]. To combat this, the government has introduced various measures, including domestic pig vaccinations [56], oral bait vaccines for wild boars [56,57], and enhanced wildlife management [56]. However, oral bait vaccines showed modest success, increasing wild boar antibody prevalence by only 12.1% [61]. Collaborative efforts of the Ministry of Agriculture, Forestry, and Fisheries and other agencies have further included fencing, surveillance, and hunting [62], as well as strict biosecurity measures [63], with vaccination belts and hygiene zones assisting to reduce transmission risks [58]. Meanwhile, PRRS outbreaks have intensified, with affected farms doubling from 34 in 2020 to 72 in 2021 [36], and despite ongoing vaccination and biosecurity efforts, the virus’s strain diversity complicates prevention. These disease outbreaks have significantly impacted pig farming, driving up medicinal expenses per pig, which rose from 2000 JPY in 2018 to 2300 JPY in 2022, a 15% increase (Fig. 7). Infrared (IR) cameras enable the automatic collection of thermal images, facilitating the observation of pigs’ huddling behavior 3 to 8 h post-vaccination, thereby offering insights into vaccination efficiency [64]. RGB (red, green, and blue) camera-based automation for monitoring heart rate, respiration rate, and postural behavior in group-housed pigs facilitates early health and welfare status detection [65], essential for disease control and industry resilience.

### Environmental issue

Offensive odors from pig farms, responsible for 65.53% of public complaints, are primarily caused by larger-scale operations [66] and are compounded by significant environmental impacts, particularly the release of GHG, for example, CH₄ and N₂O. In 2018, CH₄ emissions from pig manure management accounted for 5.06% of total livestock emissions, while N₂O represented 31.9% of emissions from manure management [67]. These emissions are exacerbated by the large volume of waste, with pig farms generating approximately 22 million tons of waste in 2017, contributing 30% of total livestock waste [66]. While solid waste is composted by pile-type and windrow-type systems, and liquid waste undergoes aerobic treatment [68], these processes, though effective at reducing odors, still contribute to GHG emissions due to the high temperatures during aerobic fermentation. Togaya et al. [69] found that conventional pig farms emit 396 kg CO2-eq/pig/year. Limited anaerobic treatment methods hinder environmental mitigation, especially when compared to practices in Europe and China [70]. Pig wastewater, rich in N₂ and organic content, is treated aerobically; however, infrastructure primarily targeting biochemical oxygen demand (BOD) [71] leaves the effluent still high in N₂, contributing to water pollution. In 2017, approximately 61% of wastewater underwent aerobic treatment, with discharge standards tightening from 600 mg N/l to 100 mg N/l [72]. The government is enhancing wastewater treatment systems and promoting Good Agricultural Practices to reduce N₂ pollution and improve sustainability in pig farming [73], although further technological upgrades are needed to meet stricter standards and reduce GHG emissions.

### Strict environmental law

Japan’s commitment to achieving carbon neutrality by 2050 [74] has led to the establishment of a robust environmental framework, including key laws, for example, the Basic Environment Law [75], the Water Pollution Control Law [76], and the Offensive Odor Control Law [77], which demands strict effluent and odor management from livestock farms. These regulations require the implementation of advanced and cost-intensive waste treatment systems. To further reduce environmental burdens, the MIDORI Strategy for Sustainable Food Systems [78] and the MeaDRI policy [79] promote innovative practices, for example, converting manure into biochar or biogas and balancing GHG reduction with the economic challenges of implementing technologies. While these regulations are critical for sustainability, they may compel farmers to invest in advanced technologies, potentially increasing production costs.

### Pork price

**Figure 8.**
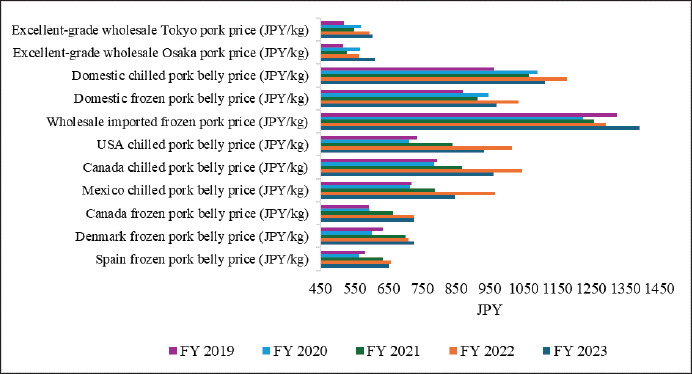
Pork prices across domestic and imported categories in Japan (Fiscal Year 2019–2023) [40]. Despite regional price disparities, domestic chilled pork belly prices exhibited a steady increase from 963 JPY in FY 2019 to 1,178 JPY in FY 2022, before experiencing a slight downturn to 1,114 JPY in FY 2023. Similarly, domestic frozen pork belly prices fluctuated, declining from 1,036 JPY to 970 JPY. Imported chilled pork belly prices fluctuated, with US prices declining in FY 2023, while Canada maintained the highest prices and Mexico the lowest. Imported frozen pork belly prices showed a steady upward trend, with Denmark leading from FY 2019–2021, Canada taking the lead in FY 2022–2023, and Spain consistently offering the lowest prices. Despite price variations, domestic pork remained costlier than imports, while chilled imports consistently outpriced frozen ones.

**Figure 9.**
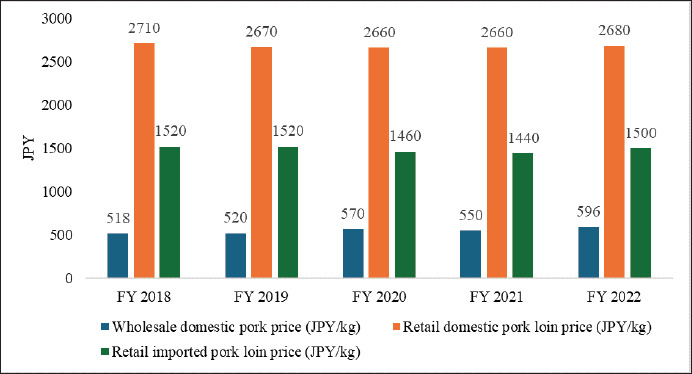
Wholesale domestic pork prices and retail pork loin prices (domestic and imported) in Japan (Fiscal Year 2018–2022) [80]. As wholesale domestic pork prices steadily rose over time, the retail prices of both domestic and imported pork loin experienced a slight decline.

Between FY 2019 and FY 2023, excellent-grade wholesale pork prices fluctuated significantly, with Tokyo’s costs rising by 16.15% and Osaka’s by 18%, reflecting regional price variations (Fig. 8) [40]. The production cost per pig, which increased by approximately 31.63% from FY 2018 to FY 2022 (Fig. 7) [30], significantly impacted wholesale domestic pork prices, climbing from 518 JPY/kg in FY 2018 to 596 JPY/kg in FY 2022 (Fig. 9) [80]. However, the retail price of domestic pork loin slightly declined from 2,710 JPY/kg to 2,680 JPY/kg, while imported pork loin prices marginally fell from 1,520 JPY/kg to 1,500 JPY/kg during FY 2018–2022 (Fig. 9) [80]. Domestic chilled pork belly prices rose steadily from 963 JPY in FY 2019 to 1,178 JPY in FY 2022, before slightly dropping to 1,114 JPY in FY 2023 (Fig. 8) [40]. Similarly, domestic frozen pork belly was adjusted from 1,036 JPY to 970 JPY (Fig. 8) [40]. Thinly sliced pork belly, favored for its rich flavor and versatility [81], drove notable price shifts in domestic pork belly. Imported frozen pork prices also rose, with wholesale prices more than double domestic prices, climbing from 1226 JPY/kg in FY 2019 to 1392 JPY/kg in FY 2022, which might have been driven by increased feed and transport expenses (Fig. 8) [40]. Imported chilled pork belly prices showed variability, with US prices declining by 8.08% in FY 2023, while Canada’s remained the highest and Mexico’s the lowest (Fig. 8) [40]. Imported frozen pork belly prices steadily rose, with Denmark leading in FY 2019–2021, Canada in FY 2022–2023, and Spain offering the lowest (Fig. 8) [40]. Despite these fluctuations, domestic prices consistently exceeded imports, and chilled imports were pricier than frozen (Fig. 8) [40]. The US-Japan Trade Agreement, set to eliminate tariffs on US chilled and frozen pork by 2027 [82], may lower prices but is likely to increase pressure on farmers, already struggling with rising production costs (Fig. 7) [30]. The government supports farmers through the ‘Pork Farm Management Stabilization Subsidy (Buta Marukin)’ [83]; however, balancing consumer needs and industry viability may require advancing technological innovation, along with traceability.

### Antimicrobial resistance (AMR)

AMR in pig farming is escalating [84], with the livestock industry accounting for 60% of antibiotics and pig farming representing 38% of that [85]. In 2018, 446.53 metric tons of antimicrobials were sold, 44.44% being tetracyclines [86], while a study in 2019 revealed 20.53 metric tons used across 74 pig farms, with penicillin dominating parenteral use (88.1%) and tetracycline accounting for 33.83% of oral usage [87]. This overuse aligns with a 256.63% increase in Methicillin-resistant *Staphylococcus aureus* cases from 2018 to 2022 [88]. Residual concentrations of antimicrobials in pig farm wastewater correlate with purchasing volumes, peaking in colder seasons [89]. Tetracycline and fluoroquinolone exhibit higher risk quotient values, signaling environmental risks [89]. Despite the National Action Plan on AMR (2016–2020) [90], persistent antimicrobial use underscores the need for stricter regulations, enhanced monitoring, and advanced waste management. Current treatment facilities remove over 80% of antimicrobial residues; however, further optimization and innovative technologies are essential to curb environmental discharge and AMR effectively [89].

### Transport welfare

With growing consumer awareness of animal welfare as a key determinant of product quality [91], studies indicate a willingness to pay more for pork sourced from pigs raised in enriched environments that ensure better welfare [92–94]. In Europe, transport duration is a critical factor in welfare labeling [95], as prolonged journeys and improper handling can contribute to physiological stress. High vehicle speeds, poor welfare indices at slaughter, extended transport distances, and frequent irregular behaviors, for example, slips, falls, and overlaps during unloading, exacerbate these stress responses, ultimately elevating cortisol and creatine kinase levels in the pig’s blood [96]. Transportation stress compromises pork quality. Sardi et al. [97] found that loin quality deteriorates when ambient temperatures exceed 22°C, transport distances surpass 26 km, travel lasts between 38 and 66 min, and more than 5.9% of pigs exhibit slips, falls, and overlaps during the time of unloading, highlighting the necessity of optimized transportation systems. To mitigate these welfare risks, precision pig farming technologies, including IoT devices [98] for real-time monitoring of transport environmental conditions and Global Positioning Systems (GPSs)-based systems [99] for optimizing vehicle speed and route planning, may offer effective solutions to minimize stress and preserve meat quality.

## Role of precision pig farming in addressing industry challenges

Developing automated and farmer-friendly animal identification systems that connect animal data to precision management systems is essential [100] as large-scale pig production grows. PLF remotely identifies and tracks the health and welfare of individual animals in real-time by analyzing tracking data, sounds, images, weight, body condition, and biological metrics [101,102]. Technologies, for example, thermal cameras, when integrated with the AI model, can accurately measure the body temperature of pigs with a remarkable precision of 97.7% [103], ensuring reliable data for health monitoring. Moreover, cameras help assess injuries, lameness, and body weight [104–106]. Oestrus detection [107] and fertility assessment [108,109] have been done using IR cameras and round-the-clock monitoring cameras.

Respiration rates and heart rates are measured by thermal IR and RGB cameras [110]. The ‘PigNet’ system leverages structural vibration sensing in pig pen floors and exhibits 90% accuracy in behavioral monitoring, addressing video-based limitations requiring constant lighting and welfare concerns with wearable sensors [111]. Moreover, the ‘PigV2 system’ utilizes ground vibration sensing to monitor pig heart and respiratory rates, achieving average errors of 3.4% and 8.3%, respectively, while ensuring non-intrusive and continuous measurement [112]. The ‘CageDot’ system employs a geophone sensor for continuous monitoring of animal heart and respiratory rates, achieving average errors of 3.8% and 8%, respectively [113]. These systems, along with a smart sensing system that has self-rejuvenation capability for device anomaly detection and automatic correction of device efficiency, ensure pig monitoring even during electricity or internet outages [114].

RFID, cameras, and environmental sensors [115] are employed in precision feeding to enhance animal health, welfare [116], and reproductive performance [117], while also reducing feed costs [118] and minimizing environmental pollution [118,119]. Facial recognition [120,121], RFID [122,123], and optical character recognition [124] are used for animal identification. ICT tools include GPS [125] and accelerometers [126] for tracking animals, RFID [123] for feeding time calculation, flow meters [127] for measuring daily water intake, and sound analyzers [128,129] for identifying respiratory problems and stress [130]. Monitoring eating and drinking behavior, including its frequency, can be achieved using deep learning algorithms [131–133] and RFID technology [134–136]. The integration of advanced sensors, for example, accelerometers, RFID tags, GPS receivers, microphones, gyroscopes, and magnetometers into wearable collar technologies revolutionizes PLF by enabling real-time monitoring of animal health and welfare while simultaneously reducing labor demands, enhancing resource efficiency, and ultimately maximizing overall productivity [137]. PLF technologies can reduce the environmental impact of livestock farming and enhance resource efficiency. Lovarelli et al. [138] conducted a study to assess the environmental sustainability of PLF technologies using life-cycle assessment (LCA) and found that the mechanical ventilation system alone reduced acidification, eutrophication, and water use by 18.60%, 19.37%, and 13.91%, respectively, and combining it with the automatic milking system led to further reductions of 23.26%, 23.04%, and 17.45%, compared to conventional farms.

Stress, respiratory diseases, and other illnesses can be identified by animal sounds [139,140]. Pig sounds, for example, coughs, screams, and grunts, serve as key indicators of pig welfare, reflecting the environmental conditions of the farm, health, and overall well-being of the pigs [141]. Indicators of respiratory diseases, for example, pneumonia, could be coughing sounds [142]. Yin et al. [129] created an AlexNet model that can identify coughing sounds with 96.8% accuracy. The audio spectrogram transformer detects abnormalities in pig vocalizations with 93% accuracy, demonstrating its potential for effective pig welfare monitoring [130]. Vocal sounds can indicate stress and reflect animal welfare status [143], however, noisy conditions in large-scale operations can limit sound-based health and welfare monitoring [144]. Recently, Wang et al. [145] developed a sound detection algorithm with 91.6% precision, tailored for noisy, large-scale pig farming operations. Animals with foodborne illnesses can be identified using sensors [146,147]. *Salmonella enteritidis* and *Escherichia coli* infected piglets can be detected early with ZigBee-based networks [148]. Microfluidics has recently become a more well-known and efficient method of diagnosing metabolic diseases (ketosis) [149]. Early disease diagnosis and treatment not only offer financial advantages but also enhance animal welfare, which is crucial for sustainability [150].

Utilizing robotic equipment for cleaning and washing farms and processing carcasses has been beneficial for saving money, labor, and time [144]. The artificial neural network can effectively predict the removal efficiency of BOD and total organic carbon in Fenton and solar photo Fenton processes used for treating pig wastewater [151]. The integration of IoT in pig wastewater treatment enhances the removal efficiencies of BOD and suspended solids [152]. By utilizing sensor-driven data, AI, and digital twins, farmers can make real-time decisions and forecast outcomes [144,153], while PLF mitigates environmental pollution and boosts production [154]. PLF assists farmers in optimizing shipping schedules [38] and monitoring pig health for disease prevention [100,150]. The efficient method of ensuring herd health is by automatically monitoring the health parameters of pigs [150].

Despite advancements in PLF technologies, their adoption is hindered by significant challenges, including costly infrastructure upgrades that make deployment expensive [154]. Additional barriers include inconsistent electricity and Wi-Fi in rural areas [155], extensive training requirements [155], concerns about accuracy [156], data ownership and cybersecurity issues [157,158], neglect of animal welfare [154], e-waste generation [159], and energy consumption [159]. These barriers highlight the gap between PLF’s theoretical potential and practical implementation, underscoring the need for holistic strategies to drive adoption. One such strategy is improving stakeholder engagement, knowledge transfer, and farmer education through social networking platforms, for example, Facebook, Instagram, and Twitter. Lamanna et al. [160] reported that 46.7% of respondents agreed that educational content on Instagram enhanced their knowledge and skills in dairy cow nutrition and management. By leveraging these platforms to disseminate PLF-related content, stakeholders can bridge knowledge gaps and promote broader adoption of these technologies. Challenges and proposed solutions for adopting PLF technologies in Japan are presented in Table 1.

## Innovations and prospects in Japan’s large-scale precision pig farming

Large-scale pig farming is transforming with the integration of precision technologies, which can reduce per capita PLF costs [161] and address the steady decline in pig farms (Fig. 6) [4]. According to the basic plan for food, agriculture, and rural areas [162], domestic pork production, based on carcass equivalent, is projected to reach 1.31 million MT by FY 2030, a 2.34% increase from 1.28 million MT in FY 2018. Innovations, for example, support vector machines for early detection of influenza virus [163], IR cameras for piglet growth monitoring [164], smart ear tags for real-time health data to identify pneumonic pasteurellosis [165], and piezo sensors and microphones for recording body-conducted sounds to detect PRRS [166] are enhancing farm efficiency. Depth cameras with Kinect v2 devices estimate body weight [167], Bluetooth tags optimize pig tracking [168], ‘PigINFO’ improves farm management, and ‘PigINFO Bio’ tackles AMR [85]. The ‘Porker’ system ensures traceability [169], and its integration with ‘AI pig cameras’ enables real-time monitoring and early disease detection [170]. ‘Digital Eyes’ [171] and ‘Hapimo P-Scale’ [172] automate weight estimation, with ‘PIG LABO’ providing advanced optimization for feed and growth analysis [173]. Semen identification and quality control are improved by wireless IC tags [22], while ‘iSperm’ streamlines semen management and artificial insemination processes [174]. Environmental sustainability is supported by BOD and pH-based intermittent aeration control systems, optimizing wastewater treatment [175]. The technology readiness level (TRL) assessment of precision pig farming innovations in Japan is presented in Table 2. These innovations contribute to a growing livestock sensor market, projected to reach $5.5 million by 2032, with a 12.48% compound annual growth rate from 2023 to 2032 [176]. Government initiatives, including the ‘Smart Agriculture Technology Catalog (Livestock)’ [22], ‘Smart Agriculture Promotion Forum 2020’ [177], ‘FY2023 Smart Agriculture Demonstration Project-Livestock’ [178], and ‘Subsidy to support farmland use efficiency (FY2020)’ [179], are driving promotion and adoption, especially in hilly areas. The government has launched the research agenda, ‘Development of management technology considering the comfort of laying hens and pigs,’ to develop lowcost management technologies that enhance production while improving animal welfare [28]. With approximately 50% of farms adopting precision pig farming technologies [24], Japan’s large-scale pig farming industry is set to enhance productivity, animal welfare, and sustainability through AI-driven solutions. Moreover, the sustainable application of PLF technology in small-scale farms, which account for over 50% of total pig farms (Fig. 1) [23], is essential to manage high feed and labor costs [30]. Shifting to non-agricultural sectors due to high production costs [30] and competition with large-scale producers may lead to a shortage of skilled labor, potentially hindering agricultural development. Therefore, this should be considered an important issue when making future policies.

**Table 1.** Challenges and proposed solutions for adopting PLF technologies in Japan.

Challenges	Proposed solutions
High costs of infrastructure upgrades	Targeting larger farms and offering financial incentives
	Low-interest credit facilities
	Tax reductions on imported PLF tools
	Domestic PLF tools production
Inconsistent electricity and Wi-Fi in rural areas	Developing rural infrastructure
Extensive training requirements	Training programs on PLF operations
	Mentorship by experienced farmers
	Livestock technology fair
	Social media learning
Validation issue	Real farm application
	Build hybrid systems using multiple algorithms
Data ownership and cybersecurity	Transparency in data handling
	Using blockchain technology
Animal welfare	Innovate humane PLF technologies
	Collaborative research among engineers and animal scientists
E-waste generation	Adopting circular production practices
Energy consumption	Optimize renewable energy use

**Table 2.** TRL assessment of precision pig farming innovations in Japan.

Innovations	Status	TRL*
Support vector machines	Trail in actual farm condition [163]	7
Infrared cameras	Trail in actual farm condition [164]	7
Smart ear tags	Trail in actual farm condition [165]	7
Piezo sensors and microphones	Trail in similar farm condition [166]	6
Depth cameras with Kinect v2 devices	Trail in actual farm condition [167]	7
Bluetooth tags	Trail in actual farm condition [168]	7
PigINFO	Fully employed [85]	9
PigINFO Bio	Trail in actual farm condition [85]	7
Porker system	Fully employed [169]	9
Porker system with AI pig cameras	Basic concept formulated [170]	2
Digital Eyes	Fully employed [171]	9
Hapimo P-Scale	Fully employed [172]	9
PIG LABO	Fully employed [173]	9
Wireless IC tags	Fully employed [22]	9
iSperm	Fully employed [174]	9
BOD and pH-based intermittent aeration control systems	Trail in actual farm condition [175]	7

^*^Technology readiness level (TRL) scale from 1 to 9, 1—basic principles observed, 2—technology concept formulated, 3—experimental proof of concept, 4—technology validated in the laboratory, 5—technology validated in a relevant environment, 6—technology demonstrated in a relevant environment, 7—system prototype demonstrated in an operational environment, 8—system completed and qualified, and 9—full deployment.

## Sustainable large-scale pig farming

**Figure 10.**
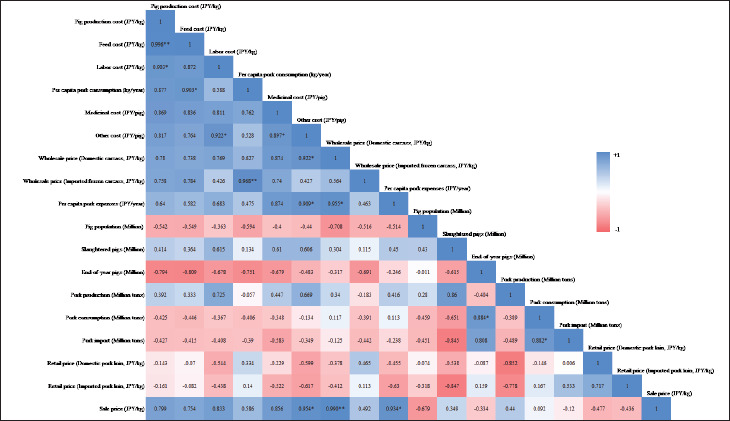
Pearson‘s correlation analysis of pig production cost with economic and production-related parameter values (* = *p* < 0.05, ** = *p* < 0.01). Pig production cost is strongly influenced by various factors, including feed (0.996), labor (0.903), medicinal costs (0.869), and other expenses (0.817), as well as per capita pork consumption (0.877).

**Figure 11.**
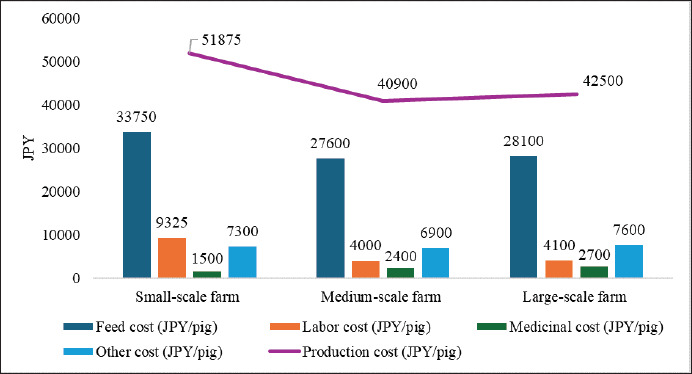
Cost distribution of Japanese small-scale (<1,000 pigs), medium (1,000–1,999 pigs), and large-scale (≥2,000 pigs) pig farming (Fiscal Year 2022). Feed, labor, and production costs per pig were lower in large-scale operations compared to small-scale ones; however, medicinal and other expenses were higher in large-scale farms. Although medium-scale operations generally incurred lower costs across most production parameters than both small and largescale farms, medicinal costs remained higher. Generated from [30].

The correlation matrix reveals that pig production cost is strongly linked to feed, labor, medicinal, and other costs, as well as per capita pork consumption (Fig. 10). The evidence indicates that increases in these factors drive up production costs, ultimately raising wholesale and retail prices for domestic carcasses and per capita pork expenses (Fig. 10). A positive correlation between production costs and imported frozen carcass prices (Fig. 10) reflects global feed price trends, driven by reliance on imported soybeans and corn and added transportation expenses. Replacing these imports with domestic rice and wheat [55] and utilizing processed food waste as feed [180] can reduce costs and promote sustainability, but proper regulations are essential. By converting food waste into nutrient-rich ‘ecofeed,’ Japan Food Ecology Center, Inc. significantly lowers pig feed costs by approximately 50% and mitigates GHG emissions by approximately 70% compared to conventional feeds made from imported grains [181]. Precision feeding can reduce environmental pollution from pig farming. One LCA study by Llorens et al. [119] revealed that precision feeding can significantly enhance the sustainability of pig farming by reducing global warming, eutrophication, and acidification potential by 7.6%, 16.2%, and 13%, respectively, compared to conventional feeding, highlighting its role in minimizing environmental pollution. Gene editing technologies, for example, clustered regularly interspaced short palindromic repeats/CRISPR-associated protein 9 (CRISPR/Cas9) and transcription activator-like effector nuclease (TALEN), can improve disease resistance [182,183] and production efficiency [184], respectively, which are permissible for the country as Japan allows gene-edited food marketing without safety evaluations if criteria are met, requiring only government notification [185]. Vertical integration in the production chain could help combat increased production costs by streamlining operations and reducing reliance on external suppliers. This concept, already implemented by NH Foods Ltd. [186] and Global Pig Farms Inc. [42], could further reduce production costs and market prices if adopted by more producers. While feed, labor, and production costs per pig are lower in large-scale farms compared to small-scale ones, medicinal and other costs per pig are higher (Fig. 11) [30]. Though medium-scale operations exhibit lower costs across all pig production parameters than small and largescale farms, except for medicinal costs (Fig. 11) [30], largescale farms benefit from a lower per capita PLF cost [161]. The number of large-scale farms is steadily increasing, while the number of medium-scale farms remains stagnant (Fig. 2) [23]. As the number of pig farms decreases and the number of pigs per farm increases (Fig. 6) [4], there is a growing opportunity to integrate precision pig farming technologies in large-scale operations, which can reduce feed [187], labor [188], and medicinal [189] costs, thus improving efficiency and cost-effectiveness.

The MIDORI strategy targets zero CO₂ emissions by 2050, with emerging technologies being developed by 2040 and fully implemented by 2050 [78]. In pig farming, integrating e-Shijisho, a digital veterinary prescription system in development [85], with technologies for precision pig farming could enhance herd health, reduce AMR, and lower medicinal costs. Renewable energy sources are crucial for operations in precision pig farming, with photovoltaic-thermal systems reducing CO₂ emissions up to 20,850 kg annually from pig farms [190]. Geothermal heat pump technology reduces electricity consumption by 38%, CO₂ emissions by 38.86%, and electricity costs by 40% in pig farms [191]. Solar energy daily saves 600 kWh of electricity in animal sheds [192], while anaerobic fermentation conserves 2865 kWh of electricity in wastewater treatment [193]. Smart IR thermal control optimizes piglet growth, reducing energy use by 36.39% in winter and 60.65% in summer [194]. This further reduces emissions and pig production costs. If renewable energy is used to operate technologies for precision pig farming, it could reduce electricity costs and, ultimately, environmental pollution. Japan prioritizes renewable energy adoption, leveraging solar, biomass, wind, and other sources for sustainable farming [195].

Farm size and environmental impact are intricately linked; while some studies indicate negative effects [196,197], others suggest that larger farms can potentially mitigate environmental pollution [198–200], through manure treatment [201]. Slurry separation, anaerobic digestion [202], and on-farm separation of liquid and solid fractions [203], along with wastewater N₂ removal by simultaneous nitrification, anammox, and denitrification [204] not only reduce GHG emissions but also produce eco-friendly fertilizers. Integrated livestock-crop systems enhance sustainability by recycling manure, improving soil fertility, and conserving resources [205]; however, overapplying animal manure to soil can exacerbate N₂, P, and K emissions into the environment [206]. The use of pig wastewater in soil and water can increase heavy metals (Zn, Cu, and Ni); however, advanced treatment processes such as flocculation, sedimentation, dissolved air flotation, Fenton oxidation, multilevel anoxic-oxic, anaerobic–anoxic–axic, oxidation ponds, and disinfection can effectively remove these metals, thereby reducing ecological risks [207]. Machine learning and deep learning algorithms can be employed in wastewater treatment to optimize P removal from effluent [208]. BODand pH-based intermittent aeration control systems in wastewater treatment save energy, reduce BOD and N₂ levels, lower electricity costs, and decrease GHG emissions [175]. Electrocoagulation technology can sustainably manage livestock wastewater by efficiently removing N₂ and P from effluent, making it an attractive alternative [209]. Gasification, pyrolysis, and anaerobic digestion enable energy recovery and create value-added products (biochar, bio-oil, and syngas) [210,211]. For example, co-digestion of pig manure with sewage sludge (30:70) boosts biogas yield [212], while with fermented liquid feed (90:10) increases CH₄ production [213]. Bioprocesses or biorefineries utilizing animal manure offer economic potential by promoting renewable energy production and sustainable manure treatment [210]. Digital twin technology, using PLF tools to create virtual replicas of physical assets and analyze real-time data to optimize farm operation, can estimate precise feed and nutrient requirements, potentially reducing food waste in the circular meat supply chain [214] and eventually lowering GHG emissions, though it remains in the early stages of development.

PLF technologies monitor individual pig health and wel-fare [215], primarily focusing on production rather than broader well-being [216]. There is controversy about animal welfare issues because of a lack of direct human-animal interaction. This shift can lead to objectification, where animals are viewed as data sources rather than sentient beings with emotional and social needs [217]. Ensuring ethical and sustainable farming requires addressing animal welfare and behavioral needs, supported by humane technologies, for example, cloud and fog computing to enhance human-animal interaction [218,219]. Financial incentives [220], tax reductions on imported tools [25], developing rural infrastructure [25], farmer training [220], technology demonstration [221], domestic tool production [28], addressing data privacy and security through blockchain technology [222], collaborative research among engineers and animal scientists [220], adopting circular production practices to reduce resource waste and e-waste [223], and regulatory frameworks are essential to optimizing PLF technologies. Pig farming is notably profitable, with incomes 2.5 times higher than beef farmers and 1.2 times higher than dairy farmers [224]. This financial advantage, combined with the increasing trend of larger farms driven by lower production costs [30], emphasizes the value of deploying PLF technologies to further enhance efficiency and sustainability.

## Conclusion

In Japan, rising per capita pork consumption coupled with insufficient domestic production has heightened reliance on imports, while declining pig farms and increasing herd sizes per farm make management more challenging. Labor shortages, escalating feed costs, GHG emissions, stringent environmental regulations, and disease outbreaks compound these issues, creating substantial barriers for farmers. Sustainable pig farming refers to practices that promote long-term viability by balancing economic profitability, minimizing environmental impact, and enhancing pig welfare. Precision pig farming technologies offer an effective solution by enabling real-time monitoring of health, welfare, and farm environments, thus optimizing production, improving animal welfare, reducing environmental impact, and lowering production costs. Several promising innovations in PLF technologies have reached the TRL 7 scale, indicating they are nearing operational deployment and demonstrating significant potential for practical application. To optimize PLF technologies, it is crucial to implement financial incentives, provide low-interest credit facilities, offer tax reductions on imported tools, promote domestic PLF tool production, and enhance data privacy and security through the integration of blockchain technology. Innovative approaches, including gene editing for disease resistance, vertical farming for increased land efficiency, renewable energy to cut costs and pollution, and using food waste as feed, contribute to sustainable practices by addressing environmental and resource constraints. Government policies, for example, offering incentives and technical facilities for deploying technologies for precision pig farming and fostering collaborative research, are crucial for developing cost-effective, user-friendly solutions. Future LCA research on large-scale precision and conventional pig farms will be critical for assessing the environmental consequences of these innovations and ensuring long-term sustainability. This holistic approach to sustainability will enable large-scale precision pig farming to play a pivotal role in advancing smart agriculture, assuring long-term food security, and trade-offs between economic efficiency, environmental impact, and pig welfare.

## List of abbreviations

CH_4_, methane; CO_2_, carbon dioxide; Cu, Copper; eq, equivalent; e-waste, electronic waste; JPY, Japanese Yen; K, potassium; kg, kilogram; kWh, kilowatt hour; mg, milligram; MT, metric ton; N_2_, nitrogen; Ni, Nickel; N/L, nitrogen per liter; N_2_O, nitrous oxide; P, phosphorus; USA, United States of America; US, United States; vs., versus; Zn, Zinc; $, United States Dollar; %, percent; °C, degrees Celsius; <, less than; ≥, greater than or equal to; :, ratio.
